# Depressive symptoms among mothers with hospitalized children in South Asia and sub-Saharan Africa

**DOI:** 10.1016/j.jad.2022.10.051

**Published:** 2023-02-15

**Authors:** Ahmed Elshafei, Julie Jemutai, Kirkby D. Tickell, Priya Sukhtankar, Amritha Bhat, Sassy Molyneux, James A. Berkley, Judd L. Walson, Pamela Y. Collins, Childhood Acute Illness and Nutrition (CHAIN) Network Investigators

**Affiliations:** a Department of Psychiatry, Larkin Community Hospital, United States of America; b Department of Global Health, University of Washington, United States of America; c Health Systems and Research Ethics Department, KEMRI-Wellcome Trust Research Programme, Kenya; d Nuffield Department of Medicine, University of Oxford, United Kingdom of Great Britain and Northern Ireland; e Department of Psychiatry and Behavioral Sciences, University of Washington, United States of America; f Clinical Research Department, KEMRI-Wellcome Trust Research Programme, Kenya

**Keywords:** Maternal mental health, Pediatric hospitalizations

## Abstract

**Background:**

Poor maternal mental health during childhood hospitalization is associated with post-discharge child mortality. We aimed to establish if maternal PHQ-9 scores during hospitalization are associated with acute stressors or longer trends in mental health status.

**Method:**

Mothers of children admitted to nine hospitals in six countries completed a PHQ-9 assessment during hospitalization and 45-days post-discharge. Community participants were recruited from homes near the hospitalized children. The prevalence and correlates of high PHQ-9 scores among hospitalized and community mothers were compared.

**Outcomes:**

Among 2762 mothers of hospitalized children, 514 (19 %) had PHQ-9 scores ≥10, significantly more than the 116 (10 %, *p* < 0·001) of 1159 community participants. Recruitment site and food insecurity were PHQ-9 correlates in both groups. Correlates of higher mean PHQ-9 scores among the hospitalized cohort included maternal illness (mean difference [MD]: 1·27, 95%CI: 0·77, 1·77), pregnancy (MD: 0·77, 95%CI: 0·27, 1·28), child HIV-infection (MD: 2·51. 95%CI: 1·55, 3·52), and lower child weight-for-height (MD: 0·21, 95%CI: 0·32, 0·11). Marriage (MD −0·92, 95%CI: −1·36, −0·48) and a positive malaria test (MD: −0·63, 95%CI: −1·15, −0·10) were associated with lower PHQ-9 scores among mothers of hospitalized children. Among mothers with PHQ-9 ≥10 during admission, 410 had repeat assessments 45-days after their child's discharge, and 108 (26 %) continued to meet the high PHQ-9 criterion.

**Interpretation:**

Among mothers of hospitalized children, there are subgroups with transient and persistent depressive symptoms. Interventions tailored to address acute stressors may improve post-discharge pediatric and maternal health outcomes.

**Funding:**

Bill & Melinda Gates Foundation OPP1131320.

## Highlights

• Mothers of hospitalized children had a higher prevalence of raised PHQ-9 scores than mothers of similarly aged children in the community.
• PHQ-9 scores among the mothers of hospitalized children were comparable to community levels 45-days after the child’s discharged from hospital.
• The correlates of PHQ-9 scores were similar across the groups, but acute stressors had more influence among the mothers of hospitalized children.

## Research in context

### Evidence before this study

Multiple clinical trials have demonstrated that lay health worker delivered mental health interventions can improve mother and child outcomes in the community. The Childhood Acute Illness and Nutrition (CHAIN) Cohort found maternal mental health to be independently associated with pediatric mortality following discharge from hospitals in six African and South Asian countries. This suggests that interventions similar to those used in the community may be valuable for families with children recovering from acute illness. To better understand maternal mental health during childhood hospitalizations, we searched PubMed on June 27th 2019, using the terms “pediatric hospitalization” and “maternal mental health” for articles published in English, French or Spanish, assessing the prevalence and aetiology of maternal mental disorders or symptoms of distress. Studies of the immediate post-partum period, i.e., prior to hospital discharge after birth, or those in exclusively from high income settings were excluded. The citations of identified studies were traced for additional relevant data. We identified two single centre studies from the same setting that suggested the prevalence of maternal mental disorders or symptoms of distress may be very high during pediatric hospitalizations, and may be correlated with the child's nutritional status (stunting and wasting), current symptoms (diarrhea), and the mother's social support. In the identified literature, it was unclear if maternal distress during pediatric hospitalization was transient and associated with acute stressors, or if it was related to a persistent mental health condition.

### Added value of this study

We included 2762 mothers whose children had been admitted to nine different hospitals in six countries and were enrolled into the CHAIN cohort. The prevalence and correlates of high PHQ-9 scores (≥10) among these mothers were compared to 1159 mothers recruited from the communities served by those hospitals. Mothers in the hospital cohort had a higher prevalence of elevated PHQ-9 scores (*N* = 514, 19 %) than mothers in the community (*N* = 116 10 %, *p* < 0·001). Among both the hospitalized and community participants, recruitment site was strongly associated with mean PHQ-9 score, as were moderate and severe household food insecurity. Acute stressors were also prominent correlates of higher mean maternal PHQ-9 during the hospitalization, including maternal illness, pregnancy, childhood wasting, and child HIV-infection, in addition to low maternal height-for-age z-score. Conversely, marriage and a positive malaria test were associated with a lower mean PHQ-9 score. The hospitalized children and their families were followed 45-days after the child left hospital, at which point 243 (11 %) of the 2310 mothers who attended this visit had high PHQ-9 scores. Among mothers who had a high PHQ-9 score during their child's admission, and also had follow-up data available, 108 (26 %) of 410 continued to meet the high PHQ-9 criterion 45 days after leaving hospital.

### Implications of all available evidence

Mothers of hospitalized children have a high prevalence of elevated PHQ-9 scores. Acute stressors are commonly associated with raised PHQ-9 scores among these mothers, but these increases have often resolved within 45 days of their child's discharge. However, nearly one-quarter of mothers had persistent depressive symptoms. This suggests inpatient pediatric stays could be used to identify mothers suffering acute and persistent mental health conditions and deliver tailored interventions designed to improve both mother and child outcomes.

## 1 Introduction

Depression is the second leading contributor to global disability, and its impact is highly gendered (GBD 2019 Diseases and Injuries Collaborators, 2020). Women of childbearing age are twice as likely as similarly aged men to become depressed (Albert, 2015). Maternal depression increases risk of suicide, a leading cause of mortality in women of child-bearing age (Rahman et al., 2013a; Haithar et al., 2018), and diminishes a mother's ability to meet her child's needs, leading to an increased risk of childhood undernutrition, impaired neurodevelopment, severe illness and mortality (Van Der Waerden et al., 2015). The link between maternal mental health and childhood morbidity suggests that the children of depressed mothers may be more likely to be admitted to hospital, and that maternal mental health may be an under-addressed determinant of child health.

The Childhood Acute Illness and Nutrition (CHAIN) Network aims to identify the biological and behavioral determinants of child mortality during and after severe childhood illness despite treatment according to existing guidelines, with a focus on mechanisms related to undernutrition (The CHAIN Network, 2017). The CHAIN cohort recruited children at admission to hospital at 9 sites in Africa and South Asia and found maternal health, including mental health, to be directly associated with mortality in the post-discharge period (The Childhood Acute Illness and Nutrition (CHAIN) Network, 2022).

In hospital, mothers typically engage with medical teams over a period of two-to-seven days, suggesting that this might be a window of opportunity to identify maternal depression, deliver brief mental health interventions and identify those needing longer-term treatment. Treating depression among mothers can improve childhood nutritional status and decrease episodes of acute childhood illness (Rahman et al., 2013b) indicating that an intervention delivered during inpatient pediatric stays could benefit both mother and child. However, the extent to which apparent depressive symptoms among mothers are associated with the acute stress of the child's hospitalization is unclear. Acute distress caused by the child's illness may be particularly responsive to brief psychological interventions, such as Problem Management Plus (PM+) (WHO, 2016), or may not require intervention beyond self-care and social support.

This analysis aims to establish if high Patient Health Questionaire-9 (PHQ-9) scores, a measure of depression, among the mothers of hospitalized children are transient, and whether they are associated with acute stressors, or reflect longer trends in maternal mental health. We compare the prevalence of high PHQ-9 scores among mothers of hospitalized children to mothers of similarly aged children in the community, determine if the correlates of high PHQ-9 scores differ between these two groups, and explore whether the elevated PHQ-9 scores observed among the mothers of hospitalized children change 45-days after the child was discharged from hospital.

## 2 Methods

The CHAIN Cohort methods are described elsewhere (The CHAIN Network, 2017). In summary, children aged 2–23 months old being admitted to Dhaka or Matlab hospitals in Bangladesh; Civil Hospital Karachi in Pakistan; Kilifi County hospital, Mbagathi District Hospital, or Migori Sub-county Referral Hospital in Kenya; Mulago Hospital in Uganda; Queen Elizabeth Central Hospital in Malawi; or Banfora Regional Hospital in Burkina Faso were eligible. Children admitted for traumatic injury, or those with an underlying condition that would require surgery within 6-months, or those who the admitting clinician felt were peri-cardiovascular arrest, were excluded. Participants were stratified by the child's mid-upper arm circumference (MUAC), aiming for a ratio of: two children with MUAC <11·5 cm if older than six-months (or <11·0 cm if younger than six-months) or with nutritional oedema, two children with MUAC ≥11·5 cm but <12·5 cm if older than six-months (or ≥11·0 but <12·0 cm if younger than six-months), and one child with a MUAC above these cut-offs.

The child's demographics, anthropometric status, clinical history, and physical examination were completed by trained healthcare providers at presentation. All children received HIV and malaria tests at admission. Within 48 h of a child's admission, trained study staff interviewed the mother to collect household and socioeconomic information, food security and diversity, and water and sanitation indicators. In addition to the mothers' characteristics, mental health assessment was carried out using the PHQ-9 tool to screen for depressive symptoms among mothers (Manea et al., 2012; Ahmad et al., 2018; Akena et al., 2013; Malpass et al., 2010; Monahan et al., 2009). The PHQ-9 scores each of the nine DSM-IV symptom criteria for major depressive disorder from “0” (not at all) to “3” (nearly every day) (Malpass et al., 2010). The PHQ-9 has been validated as a screening tool for depression in Kenya (Monahan et al., 2009), Uganda, Malawi (Udedi et al., 2018), and Pakistan (Ahmad et al., 2018). PHQ-9 scores ≥10 are considered suggestive of a higher likelihood of having a major depressive episode. To harmonize data collection, each site was trained on the administration of the PHQ-9 by the same central clinical coordinator. Mothers with high PHQ-9 scores were referred to locally available services for care. A full description of collected variables is available in Appendix 1.

Within 48 h of the child's discharge from hospital, a study fieldworker visited the family's home. The GPS coordinates of the home were recorded and further household wealth/asset assessment was conducted. Participants were followed at the study clinics for 180-days after discharge, with a repeat PHQ-9 assessment at 45-days post-discharge. Children who died during the inpatient phase did not receive a home visit and were not followed up after discharge, but a fieldworker did later collect the GPS coordinates of the village in which the family lived. During home visits, the field workers also recruited a community participant of similar age to the hospitalized child (The CHAIN Network, 2019). A nearby household was selected using a pseudo-random selection method, and families with an appropriately aged child were invited to attend the study clinic for enrolment. The enrolment procedure for these community participants were the same as the hospitalized children, but the community participants were not followed up after enrolment. We referred the subjects with PHQ-9 ≥ 10 to psychiatric services based on their country of residence and locations. We did not collect data about the implemented treatment modalities because of the limited capacity and effectiveness of those services.

### 2.1 Statistical analysis

This analysis includes only the biological mothers of the recruited children who were able to complete the PHQ-9 assessment during the child's enrolment visit. A PHQ-9 ≥10 cut off score was used for descriptive prevalence of adverse PHQ-9 scores within the sample (Kroenke et al., 2001). The PHQ-9 can also be used as a continuous scale to depict increasing severity of depressive symptoms. To maximize the power to detect correlates of adverse mental health status, the total PHQ-9 score was used as the dependent variable for linear regression models in the current analysis.

The hypothesized correlates of the initial PHQ-9 included the child's severity of illness, measured by the presence of severe inflammatory response syndrome (SIRS, binary), WHO defined respiratory distress and signs of dehydration (indicator variables: some, severe) (World Health O, 2013). A household asset index was developed using data on household structure and assets by applying principle component analysis (indicator quintiles). Household food insecurity was classified by the cumulative number of positive responses to the Food Insecurity Experience Scale (FIES, indicator variables: none, mild, moderate, severe). Other exposures of interest included maternal age (≥18 years, binary), maternal nutritional status indicated by body mass index (indicator variables: normal, underweight, overweight/obese), maternal primary employment (indicator variables: none, full-time, part time, casual labour, self-employed, dummy variables), maternal marital status (binary: marriage vs single/divorced/widowed), maternal educational level attainment (indicator variables: less than primary, primary, primary, secondary and above), child's age (months, continuous), and site of recruitment (indicator variables). The population density in the 5 km surrounding the subject's primary residence and the distance from this residence to the study hospital and the nearest medical facility were computed by mapping house GPS coordinates onto public available population density and facility data (continuous) (The Humanitarian data exchange: health facility location data for sub-Saharan Africa, Bangladesh and Pakistan, n.d.).

Univariate and multivariable linear regression analyses were applied and adjusted by site to determine the association between each variable of interest and maternal PHQ-9 cumulative score. Continuous independent variables were tested for non-linearity through inclusion of a quadratic term. Exposures of interest that were associated with PHQ-9 scores (*p* < 0·05) were then included in multivariable linear regression models. All statistical analyses were conducted in R (the R Project for Statistical Computing) and Stata 14.0 (College Station, Tx).

To understand the extent to which PHQ-9 scores during hospitalization may have been affected by acute stressors related to the child's illness, the test was repeated 45-days after discharge. Mothers whose children died between completion of the initial PHQ-9 and follow-up at 45-days, and mothers who did not attend the follow-up visit in person, were not included in the post-discharge PHQ-9 assessment. To account for the selection bias introduced by these two factors, we conducted a propensity score sensitivity analysis in which the prevalence of a positive PHQ-9 score at day 45 was adjusted for the inverse probability of attending the day-45 follow-up. This propensity score was built using variables known to be associated with mortality prior to day-45 and likely to be associated with clinic attendance at day-45 including baseline PHQ-9, child anthropometry (MUAC & HAZ), child severity of disease, household asset index, distance to facility, and site. Two further sensitivity analyses were run to assess the effect of missing PHQ data, the first replaced missing PHQ-9 at the day-45 visit with that participants PHQ-9 result from the index admission. The second imputed the missing PHQ-9 based on prevalence of an adverse score among all mothers at the baseline PHQ-9 assessment.

The CHAIN Cohort study received ethical approval from University of Oxford, University of Washington, and appropriate site-specific regulatory bodies, and is registered at ClinicalTrials.gov (NCT03208725).

## 3 Results

### 3.1 Study population

The CHAIN cohort recruited 3101 hospitalized children between November 2016 and January 2019, and 1234 community children. This analysis includes 2762 (89 %) mothers of these hospitalized children, and 1159 (94 %) mothers of community participants with available PHQ-9 data at admission. Among the included mothers of hospitalized children, 84 (3 %) were under 18 years old, and 313 (11 %) were living with HIV (Table 1). The mothers of the community children were generally comparable to the hospitalized cohort, although they were slightly less likely to be underweight or living with HIV. Among the children, those who had been hospitalized were more likely to be stunted, have a chronic medical condition, and more likely to be HIV positive in comparison to their community peers. Due to the enrolment stratification of the parent study, 1735 (63 %) of the hospitalized children had WHO defined wasting (WHZ <−2Z), while only 143 (12 %) of community children showed wasting.

**Table 1:** Participant characteristics.

	Hospitalized Cohort N = 2762	Community N = 1159
Study sites, n (%)		
Banfora	337 (12·2)	142 (12·3)
Blantyre	309 (11·2)	142 (12·3)
Dhaka	369 (13·4)	124 (10·7)
Kampala	398 (14·4)	108 (9·3)
Kilifi	220 (8·0)	141 (12·2)
Matlab	312 (11·3)	139 (12·0)
Migori	248 (9·0)	115 (9·9)
Nairobi	251 (9·1)	120 (10·4)
Caregiver characteristics		
Education level, n (%)		
None	682 (24·7)	254 (21·9)
Primary	1214 (44·0)	529 (45·6)
Secondary and above	860 (31·1)	367 (31·7)
Age groups in years, n (%)		
<18	84 (3·0)	20 (1·7)
≥18	2674 (96·8)	1138 (98·1)
Body mass index (BMI), n (%)		
Underweight	335 (12·1)	114 (9·8)
Normal weight	1633 (59·1)	663 (57·2)
Overweight	547 (19·8)	245 (21·1)
Obese	215 (7·8)	100 (8·6)
HIV infected, n(%)	313 (11·3)	101 (8·7)
No independent income, n(%)	1926 (69·7)	785 (67·7)
Unwell, n (%)	422 (15·3)	161 (13·9)
Child's characteristics		
Age groups in months, n (%)		
<6	546 (19·8)	202 (17·4)
6–12	1031 (37·3)	373 (32·2)
>12	1185 (42·9)	584 (50·4)
Male, n(%)	1558 (56·4)	618 (53·3)
Stunted, n(%)	1360 (49·2)	325 (28·0)
Wasted, n(%)	1735 (62·8)	143 (12·3)
Admission diagnosis, n(%)		
Gastroenteritis	1355 (49·1)	0 (0)
LRTI	1090 (39·5)	0 (0)
Malaria	399 (14·4)	0 (0)
Severity of illness, n (5)		
SIRS	931 (33·7)	0 (0)
Respiratory distress	827 (29·9)	4 (0·3)
Some/severe dehydration	1328 (48·10	21 (1·8)
HIV infection, n (%)	127 (4·6)	13 (1·1)
Chronic condition^a^, n (%)	70 (2·5)	2 (0·2)
Household characteristics, n (%)		
High food insecurity	366 (13·3)	145 (12·5)
Improved toilet	651 (23·6)	302 (26·1)
Improved water source	435 (15·7)	190 (16·4)
Owns livestock	1005 (36·4)	481 (41·5)

Abbreviations: HIV – Human immunodeficiency virus, LRTI – lower respiratory tract infection, SIRS – Systemic inflammatory response syndrome.

a Including sickle cell disease, thalassemia and congenital heart disease.

### 3.2 Prevalence of high PHQ-9 among participants

Overall, 514 (19 %) of the hospitalized children's mothers had positive PHQ-9 screens during the child's hospitalization. This proportion varied substantially by site, with Matlab in Bangladesh finding no mothers had PHQ-9 scores ≥10, while 96 (38 %) of mothers recruited in Nairobi had scores above that threshold (Table 2). In the community, 116 (10 %) mothers had a positive PHQ-9 screen. The prevalence of positive PHQ-9 scores was lower in the community group at each site, except for Matlab where there were also no mothers in the community above the cut-off.

**Table 2:** Prevalence of PHQ-9 scores ≥10 by site.

	Hospitalized Cohort at admission			Hospitalized Cohort 45-days post-discharge			Community representatives		
	Total N	Presumptive diagnosis		Total N	Presumptive diagnosis		Total N	Suspected depression	
		n	(%)		n	(%)		n	(%)
Banfora	337	58	(17·2)	294	24	(8·2)	142	17	(12·0)
Blantyre	309	21	(6·8)	231	11	(4·8)	142	1	(0·7)
Dhaka	369	80	(21·7)	348	47	(13·5)	124	22	(17·7)
Kampala	398	121	(30·4)	326	39	(12·0)	108	5	(4·6)
Karachi	318	21	(6·6)	270	13	(4·8)	128	7	(5·5)
Kilifi	220	37	(16·8)	192	36	(18·8)	141	22	(15·6)
Matlab	312	0	(0·0)	288	0	(0·0)	139	0	(0·0)
Migori	248	80	(32·3)	167	35	(21·0)	115	20	(17·4)
Nairobi	251	96	(38·2)	194	38	(19·6)	120	22	(18·3)
Total	2762	514	(18·6)	2310	243	(10·5)	1159	116	(10·0)

### 3.3 Correlates of PHQ-9 hospitalized participants

Site remained an important correlate of PHQ-9 score in crude and adjusted models for both the hospitalized and community groups (Table 3). In the adjusted models for the hospitalized cohort, mothers who reported being currently unwell had mean PHQ-9 scores 1·27 (95%CI: 0·77, 1·77) points higher than those who considered themselves to be well. Mothers who were stunted had a mean PHQ-9 score 0·77 (95%CI: 0·27, 1·28) higher than those with height-for-age z-score >−2. Conversely, married mothers had mean scores −0·92 (95%CI: −1·36, −0·48) lower than unmarried mothers. Among the child level correlates, HIV infection was associated with a score 2·51 (95%CI: 1·55, 3·52) points higher than HIV-negative children, while a one z-score higher weight-for-height was associated with a −0·21 (95 % CI: −0·32, −0·11) lower PHQ-9 score. Conversely, a positive malaria rapid diagnosis test was associated with a −0·63 lower score (95%CI: −1·15, −0·10). Household moderate and severe food insecurity (Mean Difference (MD): 1·33, 95%CI: 0·92, 1·74 and MD: 2·52, 95%CI: 1·96, 3·08 respectively) were associated with higher mean PHQ-9 scores in adjusted models. Higher maternal education, mothers without HIV infection, mothers who were not pregnant, child stunting, higher household wealth quintile, improved household water source and household livestock ownership were all associated with lower PHQ-9 scores in univariate models but lost significance in adjusted models. No other variables were associated with PHQ-9 at hospital admission.

**Table 3:** Correlates of aggregate PHQ-9 score during admission and among the community participants.

	Hospital cohort						Community					
	Univariate (adjusted by site)			Multivariate			Univariate (adjusted by site)			Multivariate		
	Estimate	LCI	UCI	Estimate	LCI	UCI	Estimate	LCI	UCI	Estimate	LCI	UCI
Study sites												
Banfora	Ref			Ref			Ref			Ref		
Blantyre	−2·83	−3·48	−2·19⁎	**−4·30**	**−5·11**	**−3·49**⁎	**−3·52**	**−4·38**	**−2·66**⁎	**−4·48**	**−5·40**	**−3·57**⁎
Dhaka	−0·53	−1·14	0·09	**−1·12**	**−1·97**	**−0·27**	−0·22	−1·12	0·67	−0·54	−1·60	0·53
Kampala	0·66	0·05	1·27⁎	−0·47	−1·25	0·31	**−1·98**	**−2·91**	**−1·04**⁎	**−2·81**	**−3·83**	**−1·80**⁎
Karachi	−1·95	−2·59	−1·31⁎	**−2·84**	**−3·70**	**−1·97**⁎	**−2·24**	**−3·13**	**−1·36**⁎	**−2·31**	**−3·43**	**−1·19**⁎
Kilifi	−1·09	−1·80	−0·38⁎	**−1·96**	**−2·98**	**−1·44**⁎	**−0·97**	**−1·83**	**−0·10**⁎	**−1·93**	**−2·83**	**−1·03**⁎
Matlab	−4·29	−4·94	−3·65⁎	**−4·48**	**−5·22**	**−3·74**⁎	**−4·39**	**−5·27**	**−3·53**⁎	**−4·64**	**−5·55**	**−3·74**⁎
Migori	1·83	1·14	2·52⁎	**0·74**	**0·01**	**1·47**⁎	−0·62	−1·53	0·29	**−1·78**	**−2·76**	**−0·79**⁎
Nairobi	1·78	1·09	2·46⁎	−0·19	−0·70	1·07	0·46	−0·44	1·36	−0·02	−1·08	1·05
Distance to study site (10 km)	−0·00	−0·02	0·01				−0·00	−0·02	0·01			
Distance to nearest facility (10 km)	−0·04	−0·10	0·02				0·04	−0·04	0·12			
Population density (1000/km)	0·01	−0·01	0·015				0·01	−0·01	0·02			

Caregiver's characteristics												
Education												
None	Ref						Ref					
Primary	**−0·47**	**−0·86**	**−0·08**⁎	−0·15	−0·57	0·27	**−0·58**	**−1·12**	**−0·04**⁎	−0·15	−0·69	0·38
Secondary and above	−0·58	−1·24	0·08	0·20	−0·50	0·90	**−1·26**	**−2·21**	**−0·30**⁎	−0·61	−1·56	0·34
Married	**−1·30**	**−1·75**	**−0·85**⁎	**−0·92**	**−1·36**	**−0·48**⁎	−0·45	−1·05	0·14			
Body mass index (BMI)												
Normal	Ref						Ref					
Underweight	0·08	−0·42	0·58				**0·88**	**0·14**	**1·61**⁎	0·66	−0·05	1·38
Overweight/obese	−0·28	−0·64	0·09				−0·23	−0·71	0·25	−0·06	−0·54	0·41
Stunted	**0·95**	**0·64**	**1·27**⁎	**0·77**	**0·27**	**1·28**⁎	**0·54**	**0·02**	**1·06**⁎	−0·08	−0·78	0·61
Age (>18 years)	−0·49	−1·41	0·43				1·27	−0·38	2·92			
HIV infected	**1·46**	**0·93**	**1·99**⁎	0·19	−0·51	0·89	0·82	−0·00	1·63			
No independent Income	0·24	−0·62	0·13				**−0·77**	**−1·29**	**−0·25**⁎	**−0·62**	**−1·14**	**−0·10**
Unwell	**1·77**	**1·30**	**2·24**⁎	**1·27**	**0·77**	**1·77**⁎	**0·69**	**−0·00**	**1·38**⁎	0·39	−0·28	1·05
Mother pregnant	**0·94**	**0·09**	**1·79**⁎	0·76	−0·13	1·65	−0·67	−2·19	0·85			

Child characteristics												
Age (10 months)	0·02	−0·00	0·052				0·02	−0·01	0·06			
Male	−0·19	−0·50	0·13				−0·06	−0·49	0·37			
HAZ	**−0·19**	**−0·28**	**−0·10**⁎	0·22	0·07	0·37⁎	**−0·22**	**−0·38**	**−0·05**⁎⁎	−0·20	−0·41	0·00
WHZ	**−0·18**	**−0·24**	**−0·11**⁎	**−0·21**	**−0·32**	**−0·11**⁎	−0·00	−0·07	−0·07			
Admission diagnosis												
Gastroenteritis	0·32	−0·05	0·68									
LRTI	0·15	−0·20	0·50									
Malaria	**−0·70**	**−1·21**	**−0·18**⁎	**−0·63**	**−1·15**	**−0·10**⁎						
Severity of illness												
SIRS	−0·02	−0·35	0·32									
Respiratory distress	−0·11	−0·48	0·27									
Some dehydration	0·21	−0·16	0·59									
Severe dehydration	0·39	−0·01	0·79									
HIV infected	**2·52**	**1·76**	**3·28**⁎	**2·51**	**1·55**	**3·52**⁎	0·58	−1·46	2·62			
Chronic condition^a^	0·17	−0·84	1·18				−2·10	−7·25	3·05			

Household characteristics												
Asset quintiles												
Quintile 1 (poorest)	Ref			Ref			Ref			Ref		
Quintile 2	−0·23	−0·76	0·29	−0·00	−0·55	0·54	0·25	−0·45	0·94	0·47	−0·21	1·15
Quintile 3	0·02	−0·54	0·57	0·34	−0·25	0·93	0·02	−0·74	0·78	0·40	−0·36	1·16
Quintile 4	**−0·77**	**−1·37**	**−0·18**⁎	−0·06	−0·71	0·59	**−0·84**	**−1·67**	**−0·01**⁎	−0·27	−1·12	0·57
Quintile 5 (least poor)	**−0·75**	**−1·40**	**−0·10**⁎	0·41	−0·32	1·15	**−1·53**	**−2·44**	**−0·61**⁎	−0·29	−1·25	0·67
Food insecurity												
Low	Ref			Ref			Ref			Ref		
Moderate	**1·65**	**1·27**	**2·03**⁎	**1·33**	**0·92**	**1·74**⁎⁎	**1·05**	**0·53**	**1·58**⁎	**0·90**	**0·35**	**1·44**⁎
Severe	**3·22**	**2·72**	**3·71**⁎	**2·52**	**1·96**	**3·08**⁎⁎	**2·93**	**2·25**	**3·62**⁎	**2·46**	**1·75**	**3·17**⁎
Improved toilet	−0·08	−0·51	0·35				−0·14	−0·72	0·44			
Improved water source	**−0·48**	**−0·95**	**0·01**⁎	−0·23	−0·73	0·26	0·05	−0·61	0·70			
Owns livestock	**−0·42**	**−0·81**	**−0·03**⁎	0·09	−0·32	0·50	−0·23	−0·74	0·29			

Abbreviations: HAZ – height for age z-score, HIV – Human immunodeficiency virus, KM – kilometer, LCI – lower confidence interval (95 %), LRTI – lower respiratory tract infection Ref - Reference, SIRS – Systemic inflammatory response syndrome, UCI – Upper confidence interval (95 %), WHZ – weight-for-height z-score.

a Including sickle cell disease, thalassemia and congenital heart disease.

⁎ Significant at *p* < 0.05.

### 3.4 Correlates of PHQ-9 community participants

The correlates of PHQ-9 scores were similar among the mothers of community participants. Again, site was strongly correlated with PHQ-9, as were moderate household food insecurity (MD: 0·90, 95%CI: 0·35, 1·44) and severe household food security (MD: 2·46, 95%CI: 1·75, 3·17). Mothers who did not have an independent income were also found to have 0·62 (95%CI: −1·14, −0·10) lower mean PHQ-9 scores in the community group. Mother's educational attainment, BMI, being unwell, childhood HAZ and household wealth were all associated with PHQ-9 among the community reference group in unadjusted models, but were no longer significant in the multivariate model.

### 3.5 Changes in PHQ-9 45-days after discharge from hospitals

To understand changes in depressive symptoms among mothers of hospitalized children, the PHQ-9 was repeated 45-days after discharge. Among the 2862 mothers who completed the PHQ-9 during admission, 2310 (84 %) attended follow-up 45-days after discharge, 217 (8 %) of those who did not attend had children who died during or shortly after the admission and 252 (9 %) missed the visit. The prevalence of a positive PHQ-9 screen (score of ≥10) at day-45 was similar to the baseline community prevalence across the sites, with 243 (11 %) of the mothers meeting this criterion at day-45. Of the mothers who had a positive PHQ-9 screen during admission, 410 (80 %) had available results at day-45, and 108 (26 %) continued to meet this criterion. Among the mothers who did not meet the definition during the admission, 1900 (85 %) had results at day 45, and 135 (7 %) met the PHQ-9 criteria at day-45. To assess the effect of missing data, three sensitivity analyses were conducted, including a propensity score adjustment. These secondary analyses suggested that after accounting for missing data the true prevalence of a PHQ-9 score ≥10 at the 45-day follow-up would be between 11·1 % and 12·9 % (Appendix 2), with the propensity score adjustment being at the lower end of this range.

## 4 Discussion

We found a high prevalence of elevated PHQ-9 scores among the mothers of children admitted to hospital in comparison to mothers in the community, and striking similarities between the correlates of PHQ-9 scores among both hospitalized and community groups. Most mothers who had a positive PHQ-9 screen during their child's admission had an improved mental status 45-days after discharge, and at most sites the 45-day values were close to those seen among the community representatives. This suggests that acute stressors associated with the child's hospitalization may be driving higher PHQ-9 scores during hospitalization and may resolve for the majority of mothers (79 %) during the post-discharge period. Previous analyses of these data have found maternal factors, including high PHQ-9 scores during admission, to be directly associated with post-discharge mortality (The Childhood Acute Illness and Nutrition (CHAIN) Network, 2022), suggesting that even high scores attributable to acute stressors may be a clinically significant interventional target.

In seven of the sites, the prevalence of a positive PHQ-9 screen was higher in the hospital than in the community group, but there was a considerable degree of variability between the sites. The CHAIN sites were selected to represent a variety of epidemiological settings in urban and rural environments across sub-Saharan Africa and South Asia. Considerable effort was expended to find and implement standardized, but culturally appropriate, screening tools across these facilities. However, the sensitivity of cut-off based mental health screening tools varies across cultural settings (Tandon et al., 2012), and it is likely that some of the heterogeneity between CHAIN sites reflects the varied challenges in translation of the concepts included in the PHQ-9. Of note, no mothers met the criteria for a positive PHQ-9 screen in Matlab. Previous studies in Matlab found the rate of depression in the general population to be similarly low at 0·8 % (Selim, 2010), but qualitative exploration of that data found that challenges in the adaption of the survey to this cultural setting may have been responsible for this unexpectedly low prevalence of depression (Selim, 2010). It is possible that our result also reflects the challenges in adapting the PHQ-9 to this population, as it is widely expected that major depression is a universally common mental health condition (WHO, 2021). However, the factors associated with high PHQ-9 scores were less common in Matlab. Previous CHAIN analyses have shown that children admitted to Matlab had lower illness severity scores and were more likely to be from highest wealth quintile households with low food insecurity scores. Consequently, there are reasons to believe mothers in Matlab may indeed have lower PHQ-9 scores than mothers at other CHAIN sites, rather than this solely being related to collection methods or cultural barriers to reporting mental health concerns (The Childhood Acute Illness and Nutrition (CHAIN) Network, 2022).

Interestingly, neither clinician assessed signs of severe childhood disease nor the child's diagnosis were strongly associated with the maternal PHQ-9 among hospitalized children. Mothers and clinicians may have different perceptions regarding severity of illness, or hospitalization alone may be enough to trigger significant emotional distress for mothers, and the severity of the illness at admission may have little effect above this threshold. A positive malaria rapid diagnostic test was associated with lower PHQ-9 scores, perhaps because this condition is very well understood by caregivers in malaria endemic regions. Other stressors that may be amplified during an acute childhood illness were strongly associated with PHQ-9 among the mothers of hospitalized children, including household food insecurity, childhood wasting, and maternal health status. The presence of diverse stressors during childhood hospitalization could support implementation of brief psychosocial interventions designed to help manage these challenges, such as the World Health Organization's Problem Management Plus.

Multiple studies have recognized maternal socioeconomic status (Patel et al., 2002; Abujilban et al., 2014), education, marital status, and age (Tearne et al., 2016; Muraca and Joseph, 2014) to be determinants of maternal depression. Many of these less acute factors were also associated with PHQ-9 in adjusted or unadjusted models of the mothers of hospitalized and community children. Approximately one-quarter of mothers with high PHQ-9 scores during the hospitalization did not have improved scores at day-45, indicating persisting depressive symptoms. During the child's hospitalization, it may be possible to differentiate mothers who are likely to have transient high PHQ-9 scores, and may benefit from less intensive psychological interventions designed to address stressful or adverse situations (e.g. PM+), from those mothers who will experience persistent symptoms of depression and may require therapies such as cognitive behavior therapy, group interpersonal therapy, problem solving therapy or other evidence based psychotherapies.

The correlates identified by this analysis were often highly statistically significant, but the estimated effect sizes were typically less than a 1·5 point change in the PHQ-9 score.

Nevertheless, the median PHQ-9 score among mothers of hospitalized children was 22, which represents moderately severe to severe depression. In addition to addressing the acute stressor, mothers may benefit from psychological interventions delivered during hospitalization. This would effectively capitalize on an existing interaction with healthcare services to evaluate mothers, assess and address social needs that contribute to stressors, and deliver mental health interventions that could benefit mothers and children. However, it is also important to balance the benefits of screening and brief psychological interventions among mothers of hospitalized children with the availability of resources. An ideal approach may be to customize the intensity of intervention with low intensity intervention delivered during hospitalization but stepped-up care available for those with persistent symptoms.

This study has several limitations. As an observational study, we are not able to draw causal inference from our data. Our analysis of data from day-45 gives an impression of the longitudinal trends in PHQ-9 among the mothers with hospitalized children, but we do not know the PHQ-9 scores prior to hospitalization, nor do we have access to data on the mothers' past medical and mental health history. The day-45 data had missing data, which is likely to be more common among mothers who experience mental health challenges, but our sensitivity analyses do suggest that after accounting for this missingness there still would have been a decrease in the proportion of mothers experiencing symptoms consistent with major depression.

## 5 Conclusions

Mothers of children admitted to hospital across a range of settings in sub-Saharan Africa and South Asia had worse PHQ-9 scores than their community peers. The correlates of PHQ-9 were similar in hospital and in the community, but there was a greater prominence of acute stressors, such as pregnancy, poor physical health and childhood wasting among the hospitalized group. Forty-five days after discharge from hospital the prevalence of positive PHQ-9 scores were closer to community norms, suggesting that there is a group of mothers who have transient increases in PHQ-9 scores and a subpopulation with persistent depressive symptoms. Collectively, these data suggest that approaches to maternal mental health in inpatient and post-discharge pediatric settings could be customized, beginning with low intensity mental health interventions that support caregivers through acute stressors. For mothers with persistent symptoms or major depressive episodes, stepping up treatment to include higher intensity psychotherapy and/or medication management would be appropriate. Attention to maternal mental health can improve both pediatric and maternal health and well-being.

## Funding

The CHAIN Network is supported by the 10.13039/100000865Bill & Melinda Gates Foundation [OPP1131320]. For the purpose of Open Access, the authors have applied a CC-BY public copyright license to any author accepted manuscript version arising from this submission. The funders had no role in conduct of the study, interpretation, writing the manuscript or decision to submit. No authors were paid to write this article by any company, organization or agency. All authors were not precluded from access to the data and accept responsibility to submit for publication.

## Data sharing

The CHAIN cohort data and analysis code are deposited and may be requested at: https://dataverse.harvard.edu/dataset.xhtml?persistentId=doi:10.7910/DVN/5H5X0P.

## Figures and Tables

**Figure:**
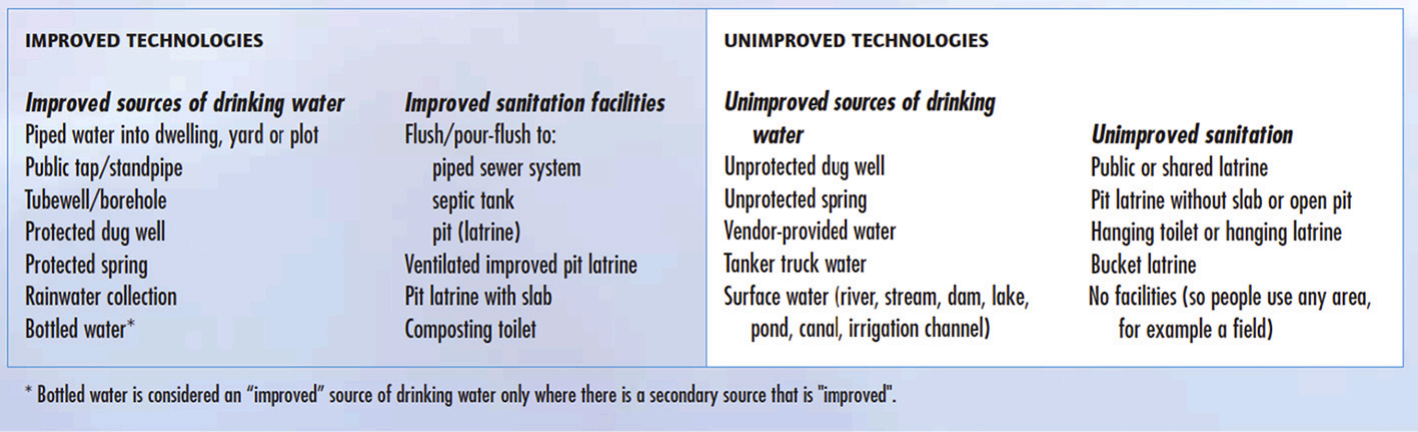


**Figure:**
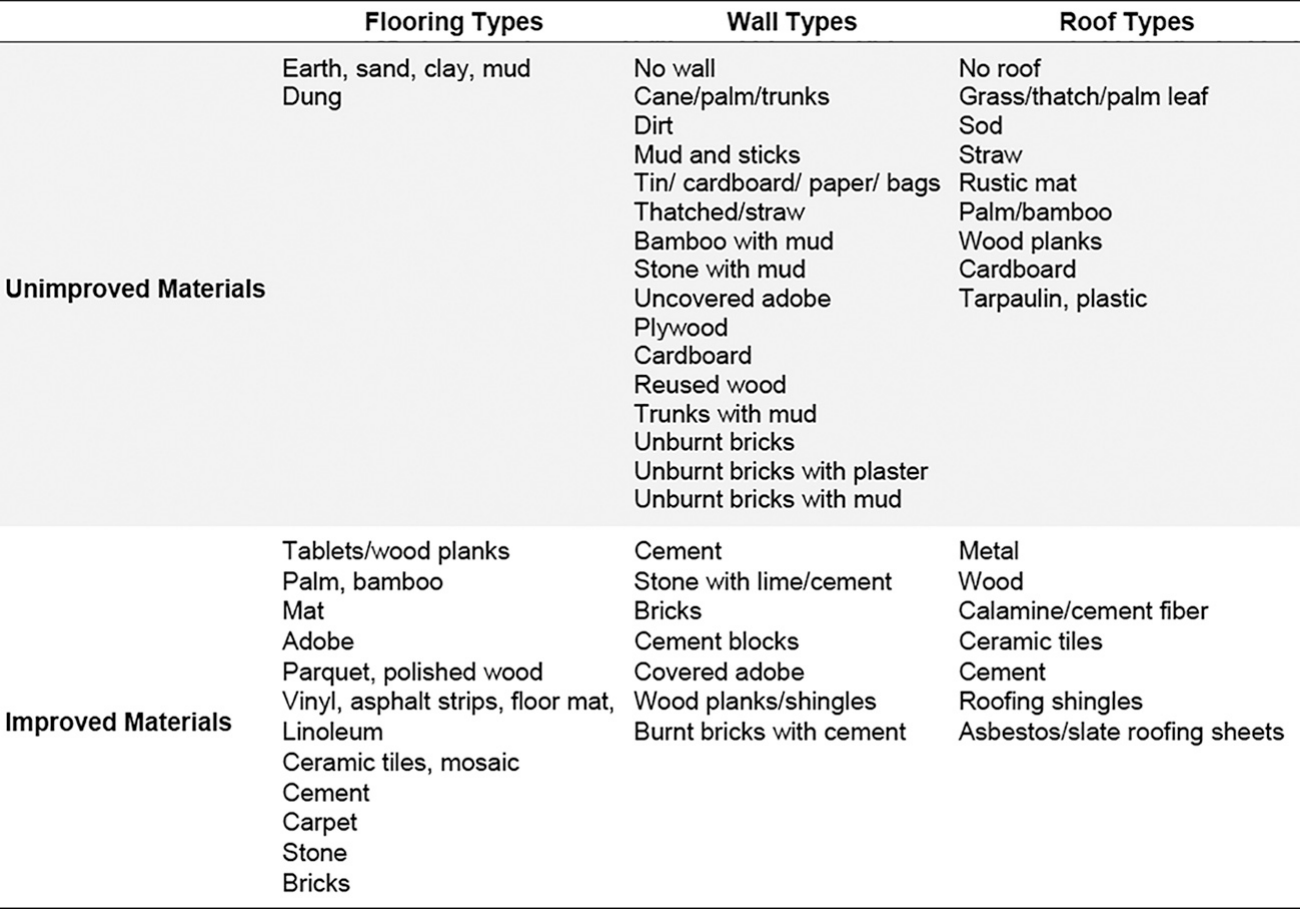


**Table:** 

Country	Source of health facility location data
Bangladesh (Matlab and Dhaka)	https://data.humdata.org/dataset/bangladesh-healthsites
Pakistan (Karachi)	https://data.humdata.org/dataset/pakistan-healthsites
African Sites (Kilifi, Nairobi, Migori, Kampala, Blantyre, Banfora)	https://data.humdata.org/dataset/health-facilities-in-sub-saharan-africa/resource/52e95479-b85a-4cbb-b9c4-8783ff0c9713

**Table:** 

	Prevalence during inpatient stay	Prevalence at day 45
Excluding missing at day 45 only	17·7 %	10·5 %
Excluding missing at both time points	17·7 %	10·5 %
Propensity score adjusted^a^	18·9 %	11·1 %
Replace missing day-45 values an individual's baseline value	18·6 %	12·6 %
Replace missing day-45 values with the mean prevalence during inpatient stay^a^	18·9 %	12·1 %

a Futher information offered below.

**Figure:**
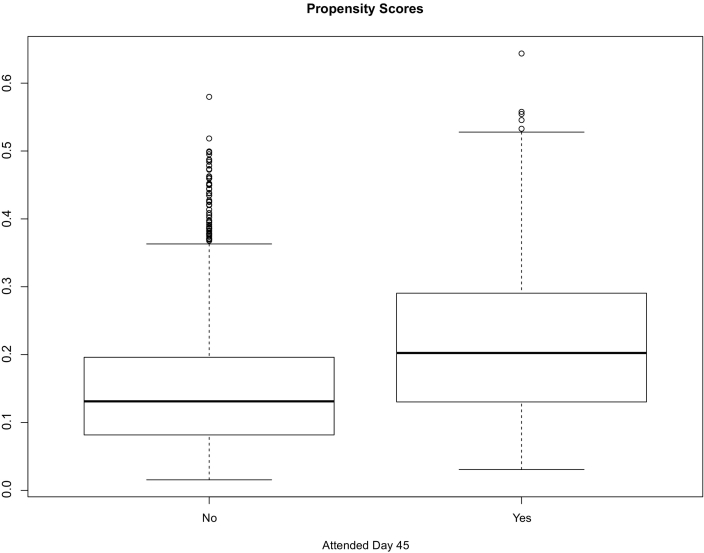


## Appendix 1 Collected variables

All CRFs and SOPs are available at https://chainnetwork.org/resources/.

*Anthropometry*

Anthropometry was performed by trained clinical assistants, including MUAC to the nearest mm using a non-stretch insertion tape (TALC, St. Albans, UK), length to the nearest mm (Seca 416 infantometer (Birmingham, UK)) and weight (Seca 825 electronic scale (Birmingham, UK) calibrated monthly) to the nearest 10 g. Caregiver MUAC utilized adult insertion tapes, weight (Seca 825 scales) and height (Seca 215 stadiometer). All anthropometric measurement z-scores were calculated using the WHO 2006 growth reference (Albert, 2015).

*Household and caregiver data*

Caregivers were interviewed during admission on their physical and mental health, social, household and access to healthcare. Physical health included their anthropometric measurements and whether the mother was also sick at the time of the interview. Caregiver's mental health was assessed using Patient Health Questionnaire 9-item (PHQ-9) tool with an additional tenth question on overall functional impairment (Rahman et al., 2013a; Haithar et al., 2018). The PHQ-9 tool has been validated and applied in LMIC contexts (Van Der Waerden et al., 2015; The CHAIN Network, 2017; The Childhood Acute Illness and Nutrition (CHAIN) Network, 2022; Rahman et al., 2013b) including when translated to local languages such as in East Africa (Rahman et al., 2013b). PHQ-9 total scores range from 0 to 27 because each of the 9 items has responses from 0 (“not at all”) to 3 (“nearly every day”). We created categories based on cut-off points at 0, 4, 9, 14 and 20 to represent screening of depression symptoms as none, minimal, mild, moderate, moderately severe and severe. We also created another variable with a single screening cut-off point of >10 to represent major depression (Rahman et al., 2013a).

A set of eight questions from Food Insecurity Experience Scale (FIES) was adapted and asked to caregivers to assess household food insecurity (WHO, 2016; Manea et al., 2012). A total score for each participant was derived by adding the responses to (yes/no) the questions. A categorical variable defining food insecurity was created with a score of 0–3, 4–6 and 7–8 defined as low, moderate and severe food insecurity respectively (Ahmad et al., 2018).

Child dietary diversity was assessed by identifying the different food groups that the child ate on a typical day. Breast milk plus 7 expected food groups (grains, roots and tubers, legumes and nuts, dairy products, flesh foods, eggs, fruits and vegetables) responses were summed to obtain total scores. Fruits and vegetables in the study were separated and not grouped in Vitamin A rich fruits and vegetables vs. other fruits and vegetables therefore combined them to form one food group.

Recommended adequate diet was defined as exclusively breastfed for children <6 months, more than or equal to two food groups and breastmilk for children 6 to 9.9 months and more than or equal to four food groups plus breastmilk for children 10 to 23 months (Akena et al., 2013).

Water hygiene and sanitation (WASH) facilities were further categorized into improved and unimproved sources based on WHO guidelines as described below (Malpass et al., 2010):

*WASH improved and unimproved sources and facilities*

Assessment of household ownership of assets such as televisions and bicycles, and housing structure were adapted from the Demographic and Health Survey (KDHS). House structure were also further categorized into improved and unimproved floor, wall and roof type as described in Lia et al. (Monahan et al., 2009) (described in the box below) and cooking fuel as defined by World Bank (The CHAIN Network, 2019). Assets and housing structure variables were then used to derive the household asset index using principal component analysis (PCA) (Udedi et al., 2018). Variables with missing data were imputed using the iterative PCA method before running PCA on complete observations (Kroenke et al., 2001; World Health O, 2013). Separate asset indices were not developed for rural and urban population. Asset quintiles were expressed in terms of quintiles with five categories depicting from the poorest to the least poor with each category representing approximately 20 % of the participants (American College of Chest Physicians/Society of Critical Care Medicine Consensus Conference: definitions for sepsis and organ failure and guidelines for the use of innovative therapies in sepsis, 1992).

*Improved and unimproved floor, wall and roof type*

Means of travel to hospital was further collapsed to a binary variable either using bus/ambulances/train/car and walking/using motorbike/rickshaw/tuktuk. Travel cost to hospital was converted to US dollars using individual country historical exchange (average) rate for each year of admission to hospital (2016–2019) (Tandon et al., 2012).

During household visit GPS coordinates of a participants was taken. Using this values Euclidian distance to the nearest health facilities and to the study hospital was calculated. Health facilities locations were mined from secondary sources such as Humanitarian Data Exchange.

A raster file with population density at 1 km^2^ was downloaded from https://www.worldpop.org/geodata/summary?id=24776. Point pattern analysis was done to extract population densities for participant using GPS coordinates.

### Clinical definitions

SIRS – Systemic Inflammatory Response Syndrome was defined in accordance with the international consensus conference on Pediatric sepsis (Selim, 2010), and includes: The presence of at least two of the following four criteria; heart rate low (<90) or high (>180)/min; temperature low (<36 °C) or high (≥38.5 °C); respiratory rate high (>34 breaths per minute) and WBC low (<5 × 109/l) or high (>17.5 × 109/l).

Severe pneumonia – Defined by using the WHO (2013) guideline; cough/difficulty breathing with either central cyanosis or oxygen saturation <90 % or lower chest wall indrawing or inability to drink/breast fed/vomiting everything or impaired consciousness (WHO, 2021).

Diarrhea – Defined by using the WHO (2013) guideline; passage of at least three loose or watery stools in a 24 h period (WHO, 2021).

Hypoglycemia and hyperglycemia were defined as blood glucose <3 mmol/L and >10 mmol/L respectively.

Malaria – Defined as positive rapid Malaria test (CareStart HRP2/pLDH).

Anemia – Defined following WHO guidelines as: none (hemoglobin >11 g/dL), mild (hemoglobin ≥10 to 11 g/dL), moderate (hemoglobin ≥7 to <10 g/dL) and severe (hemoglobin <7 g/dL) (Patel et al., 2002).

Dehydration – Was defined by the Integrated Management of Childhood Illness criteria: Some (two of: restless/irritable, sunken eyes, drinks eagerly/thirsty, skin pinch goes back slowly), severe (lethargic/unconscious, sunken eyes, not able to drink or drinking poorly, skin pinch goes back very slowly) (WHO, 2021).

Children reported to have been born with weight <2.5 kg or premature (gestational age <37 weeks) were classified as having been born small for gestational age (SGA).

Abnormal discharge was defined as leaving hospital against medical advice or absconding from hospital.

### Laboratory procedures and definitions

All children enrolled to the CHAIN cohort had HIV, Malaria and glucose rapid tests done at enrolment. Children who were known to be HIV positive as shown in the child's health record book were not tested for Malaria and HIV rapid tests. All caregivers were also offered an HIV rapid test. Sample collection area was cleaned thoroughly using an alcohol swab and dried. Blood were obtained at the time of cannulation, blood draw, through a heel or finger prick and used for the tests. All tests were done as per manufacturer's instructions while adhering to high biosafety standards and use of personal protective equipment. All test kits were used before expiry dates.

*Glucose*

OneTouch glucometer was used for glucose testing. Blood was applied on the glucometer test strip immediately after collection. The confirmation window was given time to fill up completely. The blood glucose reading was shown in the display window after 5–10 s and recorded on the Case Report Form (CRF).

*Malaria rapid test*

Malaria testing was done using CARESTART or SD Bioline Ag Pf-Pan rapid test kits depending on what was available on the site. Blood was dropped onto the round specimen well marked “S” for specimen. Four drops of diluent were added and results read after 15 min. Results were interpreted based on manufacturer's instructions for the two kits used. All invalid results were repeated using a new kit.

*HIV testing*

All the CHAIN sites used Alere 2, Determine HIV 1 and 2 or Uni‑gold HIV 1 and 2 rapid tests for HIV testing. The test was explained clearly to all care givers/participants and any positive results were kept confidential, and the family referred to the HIV service. The rapid test strip was prepared by opening and placing it on a flat surface. Blood was applied on the absorbent tip of the strip after collection. This was followed by applying 3 drops of buffer solution. Results were interpreted after 15 min. All tests were expected to have a line in the control section. If the line in the patient section was visible, then the test was interpreted as positive. If there was no line in the patient section the test was negative. The test was repeated if the results were invalid. The results were shared with the caregiver and local guidelines followed for any positive results. Positive had PCR confirmatory testing according to national guidelines.

## Appendix 2 Sensitivity analyses of day-45 missing PHQ-9s

### Propensity scores

Logistic model: Clinic Attendance = Site (indicator variables) + child muac (continuous) + child height-for-age z-score (continuous) + household wealth quintile (continuous) + child systemic inflammatory response syndrome (binary) + distance to study hospital (continuous) + carergiver cumulative PHQ-9 score at baseline (continuous).

### Impute missing values with prevalence at baseline

Unobserved cases = Missing values at day-45 ∗ Baseline prevalence = 469 ∗ 0·189 = 89

Total cases = (Imputed cases + observed cases) / N = (89 + 257) / 2862 = 0·121
